# SecM-Stalled Ribosomes Adopt an Altered Geometry at the Peptidyl Transferase Center

**DOI:** 10.1371/journal.pbio.1000581

**Published:** 2011-01-18

**Authors:** Shashi Bhushan, Thomas Hoffmann, Birgit Seidelt, Jens Frauenfeld, Thorsten Mielke, Otto Berninghausen, Daniel N. Wilson, Roland Beckmann

**Affiliations:** 1 Gene Center and Department of Biochemistry, University of Munich, Munich, Germany; 2 UltraStrukturNetzwerk, Max Planck Institute for Molecular Genetics, Berlin, Germany; 3 Institut für Medizinische Physik und Biophysik, Charité—Universitätsmedizin Berlin, Berlin, Germany; 4 Center for Integrated Protein Science Munich (CiPSM), University of Munich, Munich, Germany; Rockefeller University, United States of America

## Abstract

As nascent polypeptide chains are synthesized, they pass through a tunnel in the large ribosomal subunit. Interaction between specific nascent chains and the ribosomal tunnel is used to induce translational stalling for the regulation of gene expression. One well-characterized example is the *Escherichia coli* SecM (secretion monitor) gene product, which induces stalling to up-regulate translation initiation of the downstream *secA* gene, which is needed for protein export. Although many of the key components of SecM and the ribosomal tunnel have been identified, understanding of the mechanism by which the peptidyl transferase center of the ribosome is inactivated has been lacking. Here we present a cryo-electron microscopy reconstruction of a SecM-stalled ribosome nascent chain complex at 5.6 Å. While no cascade of rRNA conformational changes is evident, this structure reveals the direct interaction between critical residues of SecM and the ribosomal tunnel. Moreover, a shift in the position of the tRNA–nascent peptide linkage of the SecM-tRNA provides a rationale for peptidyl transferase center silencing, conditional on the simultaneous presence of a Pro-tRNA^Pro^ in the ribosomal A-site. These results suggest a distinct allosteric mechanism of regulating translational elongation by the SecM stalling peptide.

## Abstract 2

A structure of a ribosome stalled during translation of the SecM peptide provides insight into the mechanism by which the large subunit active site is inactivated.

## Author Summary

In all cells, ribosomes perform the job of making proteins. As the proteins are synthesized they pass through a tunnel in the ribosome, and some growing proteins interact with the tunnel, leading to stalling of protein synthesis. Here, we used cryo-electron microscopy to determine the structure of a ribosome stalled during the translation of the *Escherichia coli* secretion monitor (SecM) polypeptide chain. The structure reveals the path of the SecM peptide through the tunnel as well as the sites of interaction with the tunnel components. Interestingly, the structure shows a shift in the position of the transfer RNA (tRNA) to which the growing SecM polypeptide chain is attached. Since peptide bond formation during protein synthesis requires precise placement of the substrates, namely, the peptidyl-tRNA and the incoming amino acyl-tRNA, it is proposed that this shift in the SecM-tRNA explains why peptide bond formation cannot occur and translation stalls.

## Introduction

The ribosome is a large macromolecular particle that synthesizes polypeptide chains from the substituent amino acid building blocks. The active site for peptide bond formation, the so-called peptidyl transferase center (PTC), is located in a cleft on the intersubunit side of the large ribosomal subunit (reviewed by [1],[2]). As the nascent polypeptide chain is being synthesized, it passes through a tunnel within the large subunit and emerges at the solvent side, where protein folding occurs. Recently, nascent polypeptide chains have been directly visualized within the ribosomal tunnel extending from the PTC to the exit site on the back of the large subunit [3]–[5], as originally predicted by Lake and coworkers in the 1980s [6],[7]. The X-ray structures of bacterial and archaeal ribosomes have revealed that the ribosomal tunnel is predominantly composed of ribosomal RNA (rRNA) [8]–[12], consistent with an overall electronegative potential [13],[14]. In addition to rRNA, the extensions of the ribosomal proteins L4 and L22 (L17 in eukaryotes) contribute to formation of the tunnel wall, and form a so-called constriction where the tunnel narrows [8],[9]. Near the tunnel exit, the bacterial-specific extension of L23 (L25 in eukaryotes) occupies a similar position to the r-protein L39e of eukaryotic and archaeal ribosomes [10]–[12].

Despite its universality, a functional role for the ribosomal tunnel is only beginning to emerge. For many years, the ribosomal tunnel was thought of only as a passive conduit for the nascent polypeptide chain; however, accumulating evidence indicates that, for some nascent chains, the tunnel plays a more active role (reviewed by [15]). In particular, a number of leader peptides have been identified that induce translational stalling in response to the presence or absence of an effector molecule, and in doing so regulate translation of a downstream gene (reviewed by [16],[17]). Well-characterized examples include the eukaryotic arginine attenuator peptide (AAP) and cytomegalovirus gp48 uORF, as well as the bacterial ErmC, TnaC, and SecM leader peptides, for which mutations in the leader peptide sequences, or within the ribosomal tunnel components, can relieve the translational arrest [18]–[21]. The implication of a direct interaction between specific residues of the leader peptide with distinct locations of the ribosomal tunnel has been confirmed by a recent cryo–electron microscopy (EM) and single particle reconstruction of a ribosome stalled during translation of the TnaC leader peptide by the presence of high concentrations of free tryptophan [4].

**Figure 1:**
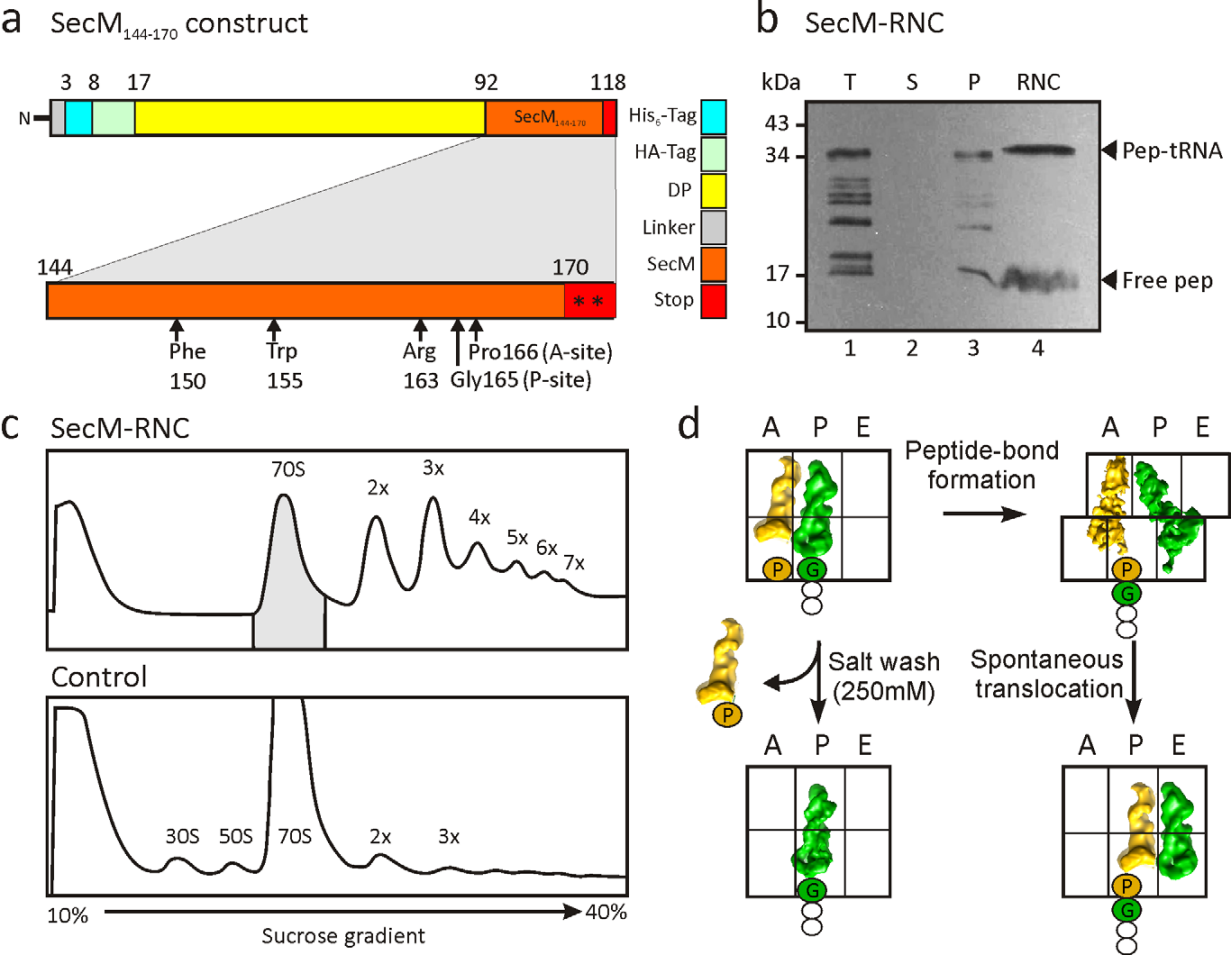
Generation of SecM-stalled RNCs. (A) Schematic showing the construct used for translation. The SecM stalling window (residues 144–170, orange) was inserted after the DP sequence and flanked by tandem stop codons (asterisks). Critical residues for stalling are marked by arrows. (B) Western blot (using anti-HA antibody) of SDS-PAGE of the translation reaction (T, lane 1), supernatant (S, lane 2), and pellet (P, lane 3) fractions following centrifugation, as well as the purified RNC following affinity column (RNC, lane 4). The position of the peptidyl-tRNA (pep-tRNA) and free SecM peptide (free pep) are indicated. (C) Sucrose-gradient profiles of the SecM RNCs (after Co-NTA purification) (upper panel) and control translation extract without template (lower panel). The shaded 70S monosomes were collected and pelleted for cryo-EM reconstruction. (D) SecM stalling occurs with SecM-tRNA^Gly^ located at the P-site of the ribosome and Pro-tRNA^Pro^ at the A-site [20],[23]. During purification of the SecM RNC, the Pro-tRNA^Pro^ in the A-site can dissociate because of high salt washing, or undergo slow peptide bond formation [23] and form a ratcheted hybrid state [31]–[34],[53] with SecM-Pro-tRNA^Pro^ in the A/P-site and deacylated tRNA^Gly^ in the P/E-site. The hybrid state may spontaneously translocate, albeit slowly [54],[55], to form an unratcheted post-state with SecM-Pro-tRNA^Pro^ in the P-site and deacylated tRNA^Gly^ in the E-site.

In contrast to stalling by TnaC, translational stalling by SecM does not require an effector molecule [22]. A minimal stalling sequence comprising 17 amino acids (aa) (SecM_150–166_) of the 170-aa SecM leader peptide is sufficient to induce translational arrest [20]. Furthermore, unlike with TnaC, where stalling occurs naturally at the UGA stop codon, i.e., during termination [19], stalling of SecM occurs during elongation at a CCU sense codon (encoding Pro166) [20]. The stalled complex has the peptidyl-tRNA (SecM-tRNA^Gly^) at the P-site and Pro-tRNA^Pro^ at the A-site of the ribosome [23], and is thus stalled in a pre-translocation state prior to peptide bond formation. Yet, transfer of the SecM nascent peptide from the tRNA^Gly^ to the tRNA^Pro^ can still occur slowly [23], and is triggered by the presence of SecA activity to alleviate stalling [24]. Mutational analysis has identified the conserved Arg163, Gly165, and Pro166 of SecM as being critical for translational stalling [20],[25], with additional contributions from Phe150, Trp155, Ile156, Gly161, and Ile162 [20] (Figure 1A). Translational arrest is also alleviated by modification of ribosomal components of the tunnel, namely, mutation A2058G, A2062U, or A2503G, or single adenine insertions at A749–A753 of the 23S rRNA [20],[26],[27], as well as mutations, insertions, or deletions within ribosomal proteins L22 and, with lesser effect, L4 [20],[26]. Despite extensive biochemical characterization, the mechanism by which the PTC of the ribosome is inactivated remains unclear. One structural study on SecM stalling at low resolution purported that the elongation arrest arises from a cascade of rRNA conformational rearrangements [28].

Here we have determined a cryo-EM reconstruction of a SecM-stalled ribosome nascent chain complex (RNC) at 5.6 Å, enabling the direct interaction between critical residues of SecM and the ribosomal tunnel to be visualized. While we find no evidence for a cascade of rRNA conformational changes, we observe a shift in the position of the tRNA–nascent peptide linkage of the SecM-tRNA. This shift moves the carbonyl carbon of the SecM-tRNA away from the A-tRNA and, thus, is likely to contribute to the impaired activity of the PTC, explaining the SecM-mediated translational arrest.

## Results/Discussion

### Cryo-EM of SecM-Stalled RNCs

**Figure 2:**
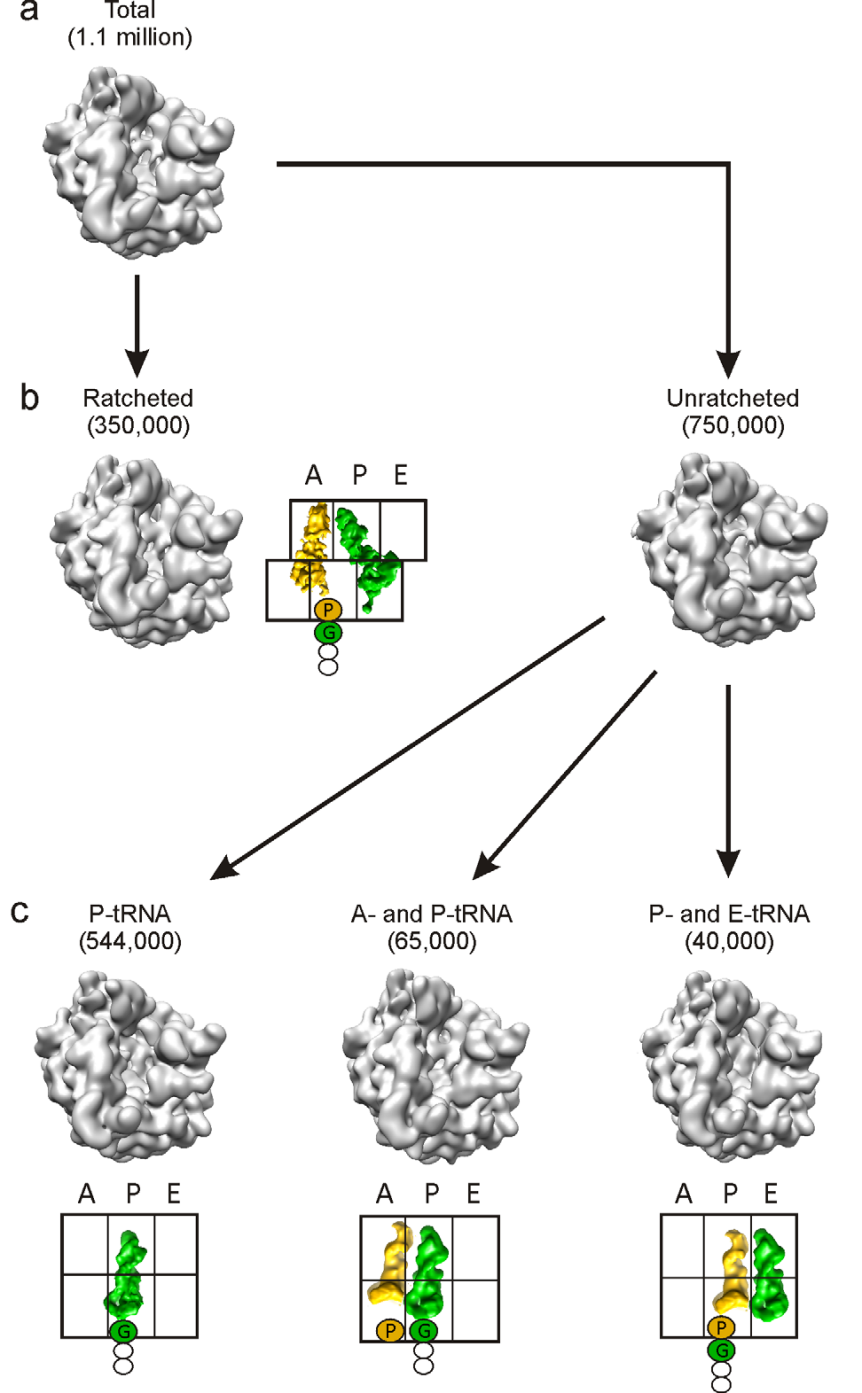
Schematic for in silico sorting of the SecM RNCs. The unsorted volume (A) containing a total of 1.1 million particles with density in all three tRNA binding sites was initially sorted into two populations (B) based on the ratchet-like subunit rearrangement of the small subunit relative to the large subunit. The ratcheted population (350,000 particles; 32%) had tRNAs present in A/P- and P/E-sites, whereas the unratcheted population (750,000 particles; 68%) could be further sorted into three subpopulations (C): a dominant fraction (544,000 particles; 73%) with P-tRNA only, and two minor fractions with A- and P-tRNAs (65,000; 12%) and with P- and E-tRNAs (40,000; 7%).

**Figure 3:**
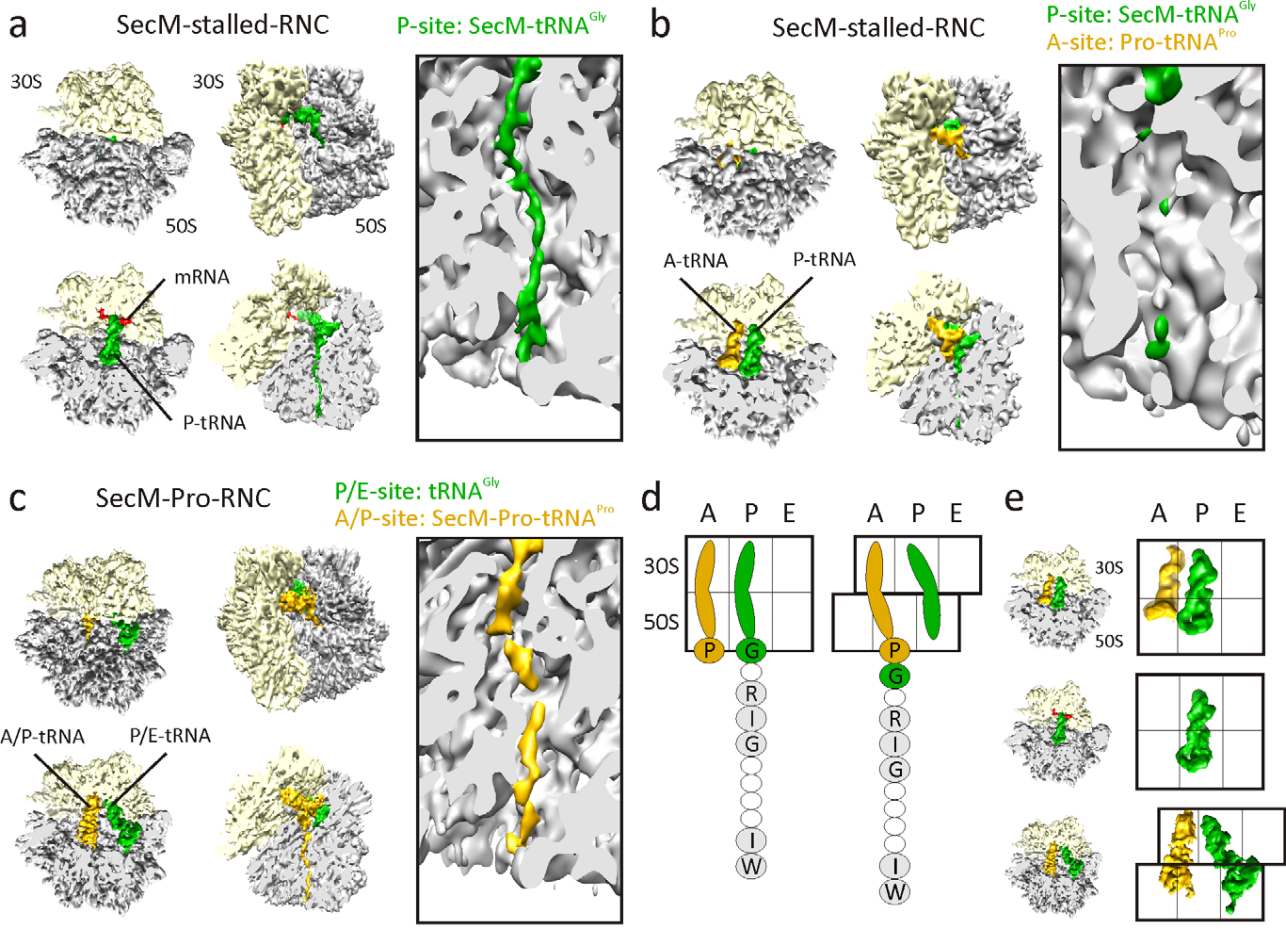
Cryo-EM reconstructions of SecM RNCs. (A–C) Cryo-EM reconstructions of (A) SecM-stalled RNC with SecM-tRNA^Gly^ (green) in P-site, (B) SecM-stalled RNC with additional Pro-tRNA^Pro^ (gold) in the A-site, and (C) SecM-Pro-RNC, with SecM-Pro-tRNA^Pro^ (gold) in A/P-site and tRNA^Gly^ (green) in P/E-site. For each reconstruction, the top two diagrams show a top and factor view of the small (30S, yellow) and large (50S, gray) subunits, with respective cross-sections below. Right-hand panels show close-up of the tunnel views of each complex. (D) Schematic showing unratcheted SecM-stalled state (left), with Pro-tRNA^Pro^ in the A-site and SecM-tRNA^Gly^ in the P-site, and post-arrest ratcheted state (right), with SecM-Pro-tRNAPro in the hybrid A/P-site and tRNA^Gly^ in the P/E-site. Residues important for SecM stalling are shaded and labeled with single-letter amino acid code. (E) Schematic showing the relative positions of the tRNAs from the complexes in (A–C).

To generate SecM-stalled RNCs, a construct was prepared that encodes consecutive His- and HA-tags connected by a linker region to the C-terminal 27 aa (SecM_144–170_) of SecM (Figure 1A). The SecM-stalled RNCs were generated using an *Escherichia coli* in vitro translation system and purified using Co-NTA affinity chromatography as described previously (Figure 1B) [29]. To ensure homogeneity of the RNC sample, 70S monosome fractions of the SecM-stalled RNCs were separated from affinity-purified polysome fractions using sucrose density gradient centrifugation (Figure 1C). An initial cryo-EM reconstruction was generated from 1.1 million particles of the monosome fraction, revealing a 70S ribosome with tRNAs occupying A-, P-, and E-sites, very similar to that previously reported [28]. Previous biochemical analysis has shown that the majority of ribosomes stall at position 165 of the SecM ORF, with a glycine as the most C-terminal amino acid bound to the peptidyl-tRNA in the ribosomal P-site, and an obligatory Pro-tRNA^Pro^ in the A-site. An additional minor fraction of ribosomes undergo slow transfer of the nascent peptide from the tRNA^Gly^ to the tRNA^Pro^ after longer incubation times (Figure 1D). We therefore applied an in silico sorting procedure [30] to resolve the conformational heterogeneity within the complex (Figure 2). Of the 1.1 million particles sorted, the largest fraction (750,000 particles) had unratcheted ribosomes, with the majority (544,000 particles; ∼50%) containing a single peptidyl-tRNA at the P-site. This state was reconstructed at 5.6 Å (0.5 Fourier shell correlation [FSC]; Figure S1) and termed the SecM-stalled RNC (Figure 3A). At this resolution, clear density for the SecM nascent polypeptide chain is observed within the exit tunnel of the large subunit (Figure 3A).

As expected, a subpopulation of P-tRNA containing unratcheted ribosomes with an additional A-tRNA was also observed, representing SecM-stalled RNCs with Pro-tRNA^Pro^ still bound in the A-site. Partial dissociation of the A-site tRNA during the high salt (250 mM KOAc) wash protocol in our RNC preparation may provide an explanation for the low overall occupancy of A-site-bound Pro-tRNA^Pro^ (9%) (Figure 2). Despite low particle numbers, we were able to reconstruct this complex to a resolution of 9.3 Å (Figure S1); however, the limited resolution does not allow for the direct visualization of the SecM nascent chain (Figure 3B). There is, however, no conformational difference between the two SecM-stalled RNCs, indicating that the presence of the Pro-tRNA^Pro^ in the A-site does not trigger any large-scale conformational changes related to stalling (Figure S2).

Computational sorting revealed that another subpopulation (350,000 particles; 32%) of ribosomes had undergone a ratchet-like subunit rearrangement of the small subunit relative to the large subunit (Figure 2). The reconstruction of the ratcheted complex at a resolution of 6.0 Å revealed two tRNAs present in A/P and P/E hybrid sites and clear density for the nascent chain in the tunnel (Figure 3C). This peptidyl-tRNA observed in the A/P hybrid site is in accordance with the biochemical studies demonstrating that with incubations longer than 60 min, such as in the RNC purification protocol used here, there is a slow release from the arrested state [23], i.e., transfer from tRNA^Gly^ in the P-site to the A-site-bound Pro-tRNA^Pro^ (Figures 1D and 3D). Following peptidyl transfer, ribosomes are free to ratchet and the associated tRNAs can adopt hybrid states [31]–[34] (Figure 3D). On this basis, we interpret the ratcheted complex as a post-arrest ribosome containing SecM-Pro-tRNA^Pro^ in the A/P-site and deacylated tRNA^Gly^ in the P/E-site, and thus termed it SecM-Pro-RNC (Figure 3C). The SecM-Pro-RNC hybrid state is similar, in terms of degree of ratcheting, tRNA positions, and L1 stalk movement, to that observed previously with 70S ribosomes containing peptidyl-tRNA mimics fMetLeu- or fMetTrp-tRNA [33],[34] (Figure S3).

### Visualization of the SecM Nascent Chain within the Ribosomal Tunnel

A molecular model for the SecM-stalled RNC was built by rigid-body docking of the ribosomal subunits from the model of the TnaC-stalled RNC [4]. Within the limits of the 5.6-Å resolution, we observe an excellent agreement between the ribosome structures of SecM-stalled RNC and TnaC-stalled RNC [4], as well as with the crystal structures of bacterial ribosomes [11],[12]. We find no evidence for any cascades of rRNA conformational rearrangements as proposed earlier [28], suggesting that the purported rearrangements may have arisen due to conformational heterogeneity, which we also observed in the unsorted SecM-stalled RNC sample (Figures 2 and S2). Taken together, in silico sorting of our dataset resulted in segregation into subpopulations with defined functional/conformational states (Figures 2, 3E, and S2) that are in agreement with the biochemical data. Moreover, this procedure allowed higher resolution reconstructions to be obtained, enabling the nascent polypeptide to be directly visualized within the ribosomal tunnel, which is not possible at lower resolutions (Figure S4).

**Figure 4:**
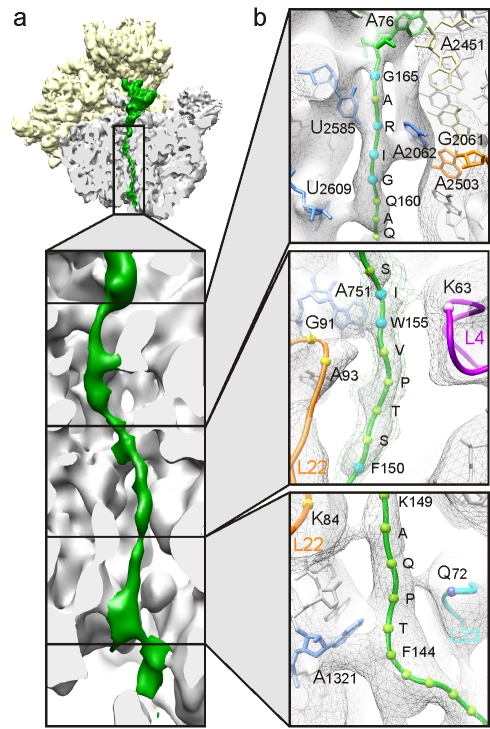
SecM nascent chain interactions with tunnel components. (A) Cross-section of the large ribosomal subunit of the SecM-stalled RNC revealing the sites of interaction between the SecM nascent chain (green) and the ribosomal tunnel (gray). (B) Close-up of the upper, middle, and lower regions of the ribosomal tunnel with density (gray mesh) and molecular models for SecM nascent chain (green, with balls marking the Ca of the labeled residues; blue indicates the residue is important for stalling), the 23S rRNA (gray, except for selected colored nucleotides), and ribosomal proteins L4 (purple), L22 (orange), and L23 (cyan).

The density characteristics indicate that the SecM nascent chain adopts a predominantly extended conformation, similar to that of TnaC [4] (Figure S5), but with some slight compaction in the upper tunnel (Figures 4 and S6). A large region of compaction is observed near the tunnel exit, as reported previously for TnaC and Helix RNCs [4],[5], but the distance from the PTC indicates that this region is unrelated to the SecM sequence in our construct. Nevertheless, a compacted conformation for SecM between residues 135 and 159 has been reported based on fluorescence resonance energy transfer measurements [35], which would encompasses SecM in the lower tunnel region. Thus, based on an essentially extended conformation of the SecM nascent chain in the critical region, we have built a polyalanine model that has been used to interpret the observed contacts of SecM with components of the ribosomal tunnel (Figure 4; Table S1). Because the resolution of the map is limited to approximately 6 Å, all analysis was restricted to the proximity of the Cα atoms of SecM.

### Interaction of the SecM Nascent Chain with Components of the Ribosomal Tunnel

In the upper region of the tunnel of the SecM-stalled RNC, three connections are observed between the nascent chain and components of the tunnel wall, namely, 23S rRNA nucleotides U2585, U2609, and A2062 (Figure 4). Strong density connects A2062 to the proximity of Arg163 of SecM. This contact is likely to be critical for SecM stalling since scanning mutagenesis with Ser indicates that mutation of only Arg163 of SecM abolishes SecM stalling [20],[25]. Similarly, the mutation A2062U abolishes both SecM and ErmC stalling [27]. A2062 is highly flexible [36] and appears to adopt a position flat against the tunnel wall in the SecM-stalled RNC, possibly constrained by the close proximity of the bulky Arg163 and Ile162 residues of SecM. Consistent with this, Vazquez-Laslop et al. [27] have recently suggested that this orientation of A2062 triggers a relay through A2503 (which is also essential for SecM and ErmC stalling [27]) to inactivate the PTC. In contrast, the interaction of U2585 with SecM in the proximity of Ala164, and of U2609 with the slightly compacted _160_QAQ_158_ area of SecM, are less likely to be important for SecM stalling (Figure 4), since mutations of these amino acid residues do not significantly affect SecM stalling [20],[25].

Within the constriction located in the mid-tunnel region, only one major contact is observed to SecM, namely from the vicinity of A751 towards Trp155/Ile156 of SecM (Figure 4). Insertion of adenine within the five consecutive adenines A749–A753 of the 23S rRNA, or either mutation Ile156Ala or Trp155Ala, abolishes *E. coli* SecM stalling [20]. Furthermore, mutations of the neighboring ribosomal protein L22, specifically Gly91Ala and Ala93Ser at the tip of the β-hairpin that interacts with A751, also suppress translation arrest due to SecM [20],[26], as well as TnaC [37]. Interestingly, TnaC also encodes a tryptophan (Trp12) that is located in a similar position in the tunnel constriction, but which establishes an apparently different interaction with the tunnel that involves directly the loop of L22 as well as A751 (Table S1) [4]. Deeper in the tunnel, the nascent chain establishes contact with K84 of L22 and Q72 of L23, but predominantly with helix 50 (H50) of the 23S rRNA in the proximity of A1321 (Figure 4). This region of SecM is poorly conserved and not essential for stalling; however, we note that SecM_150–166_ is less efficient at stalling than SecM_140–166_ [20], consistent with a fine-tuning role of these residues in the placement of the critical Arg163 [25].

### Perturbation at the PTC of the SecM-Stalled RNC

**Figure 5:**
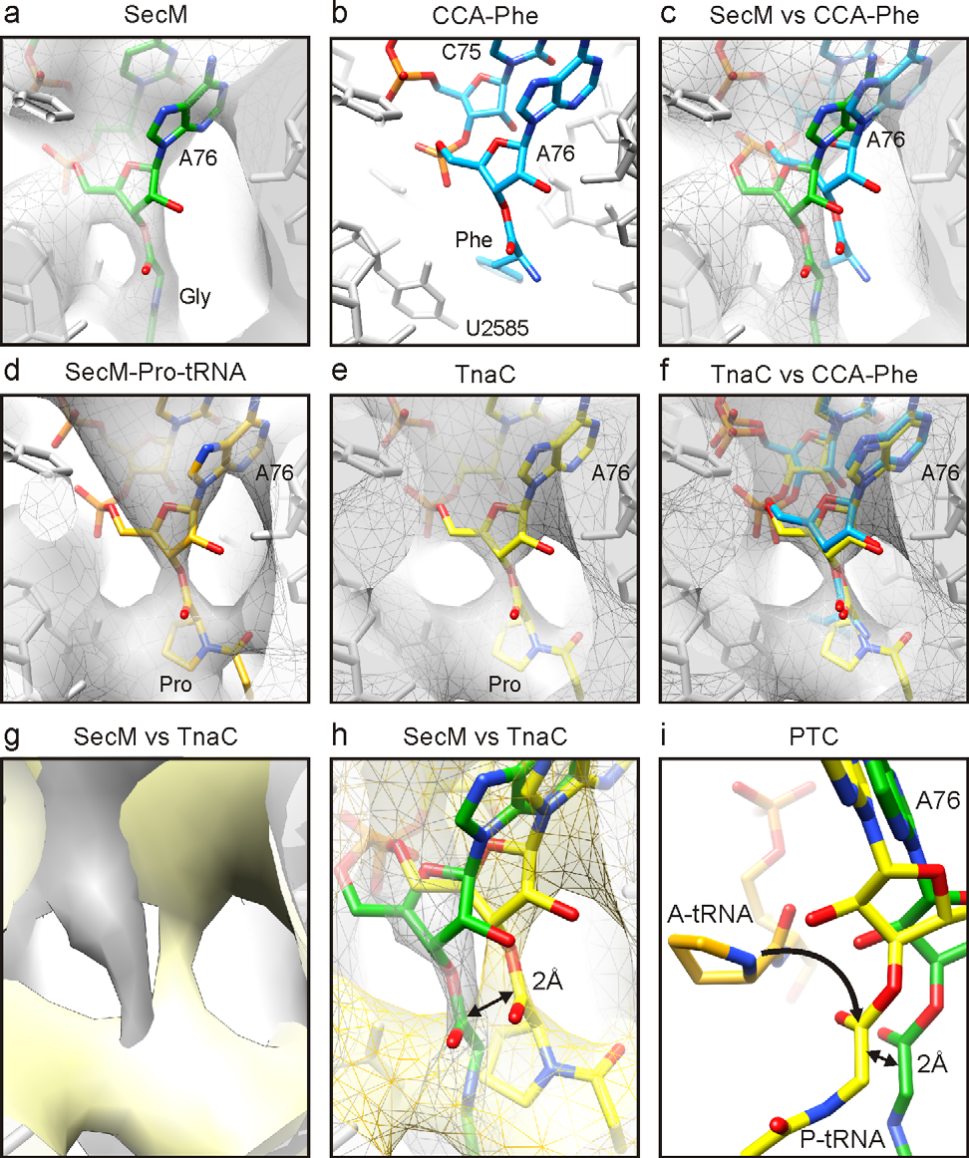
Inactivation of the ribosomal PTC by SecM stalling. (A–C) Identical views of the position of (A) the SecM-tRNA^Gly^ (green) in the map (gray mesh) of the SecM-stalled RNC and (B) CCA-Phe (cyan) based on the crystal structure of the archaeal large ribosomal subunit in complex with CCA-PCB (PDB ID1VQN) [39]. (C) Comparison of (A) and (C). (D–F) Identical views of the position of (D) Pro-tRNA^Pro^ (orange) in the map (gray mesh) of the SecM-Pro-RNC and (E) TnaC-tRNA^Pro^ (yellow) in the map (gray mesh) of the TnaC-stalled RNC. (F) Comparison of (B) and (E). (G and H) Comparison of the SecM- (gray) and TnaC-stalled RNC (yellow) maps as surfaces (G) and as mesh with molecular models for SecM-tRNA^Gly^ (green) and TnaC-tRNA^Pro^ (yellow) (H). (I) Position of Pro-tRNA^Pro^ (A-tRNA, orange; derived from PDB ID1VQN) [39] relative to TnaC-tRNA^Pro^ (yellow) and SecM-tRNA^Gly^ (green). The arrow indicates the nucleophilic attack of the α-amino of the A-tRNA on the carbonyl carbon of the P-tRNA, which is displaced by 2 Å in the SecM-stalled RNC.

**Figure 6:**
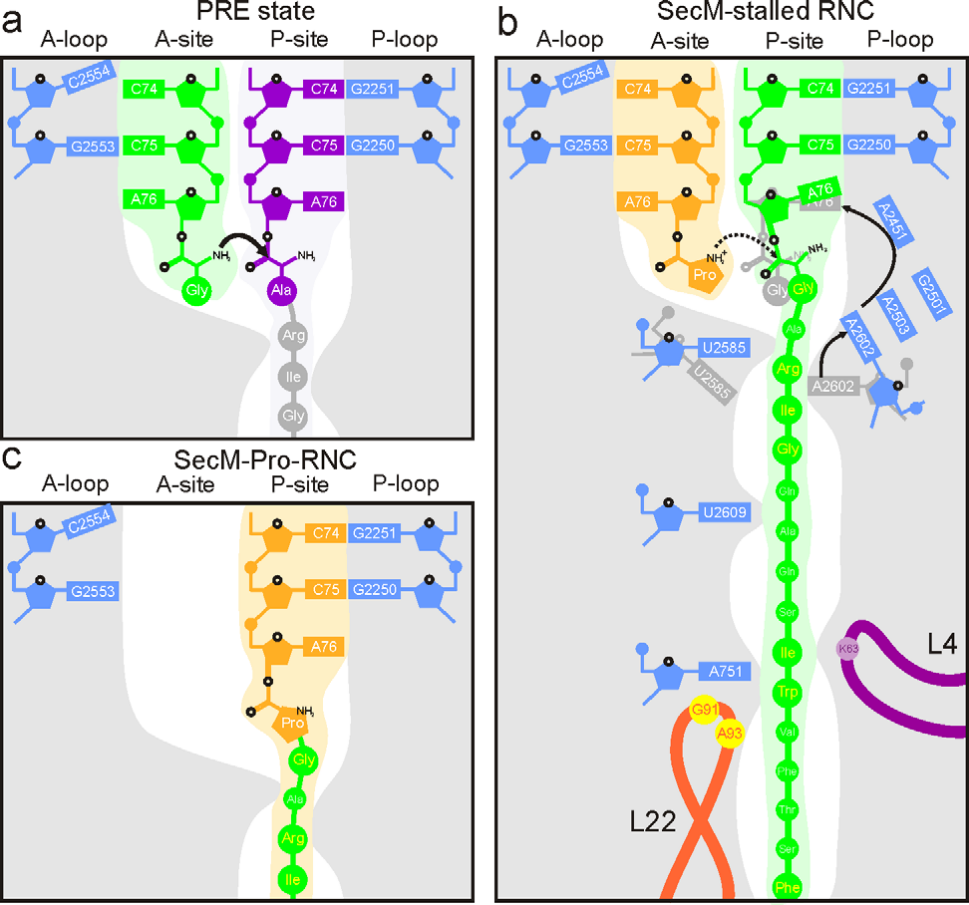
Schematic for SecM action. (A) Schematic representation showing canonical peptide bond formation where the CCA-ends of the tRNAs are precisely positioned to promote nucleophilic attack of the carbonyl carbon of the P-tRNA (purple) by the α-amino of the A-tRNA (green). (B) Interaction of the SecM nascent chain with components of the tunnel aids in the positioning of the critical Arg163, which interacts with A2062 of the 23S rRNA. Interaction of A2062 with A2503 has been proposed to trigger a relay that leads to inactivation of the PTC [27]. We propose that this results from a shifted position of the A76 of the SecM-tRNA^Gly^ in the P-site, which prevents efficient attack of the A-tRNA. (C) During prolonged SecM stalling, or by SecA activity, release from the arrested state occurs. The SecM-Pro-tRNA^Pro^ forms through peptide bond formation and can now adopt an A/P hybrid state.

At the PTC, density for the ester linkage between the nascent chain and the terminal A76 of the P-tRNA is clearly observable in the SecM RNC map (Figure 5A). The location of the CCA-end of the P-tRNA is also well characterized from a multitude of ribosomal crystal structures and is essentially identical regardless of whether CCA-end mimics or P-tRNAs are bound to bacterial 70S ribosomes or archaeal 50S subunits [12],[38],[39] (Figure 5B). Therefore, we were surprised to find that the peptide ester linkage associated with the terminal A76 appears to be shifted in the SecM-stalled RNC, relative to the crystal structures (Figure 5C). In contrast, the position of the CCA-end of the SecM-Pro-tRNA (Figure 5D), as well as that of the TnaC-tRNA [4] (Figure 5E), is not shifted compared to the crystal structures (Figure 5F). Although chloramphenicol was added to reduce peptidyl-tRNA hydrolysis [40], it is unlikely that it had an effect on the P-site peptidyl-tRNA [41], since the shift is not seen in the SecM-Pro-tRNA (Figure 5D), nor in a reconstruction of an *E. coli* RNC with a non-stalling peptide (Figure S7), both of which were also purified in the presence of chloramphenicol. A direct comparison of the density maps (Figure 5G) and models (Figure 5H) for the SecM- and TnaC-stalled RNCs [4] suggests that the A76 ester linkage has shifted by approximately 2 Å. Peptide bond formation requires precise positioning of the A- and P-tRNAs to orient the α-amino group of the A-tRNA for nucleophilic attack on the carbonyl carbon of the P-tRNA [2],[39] (Figures 5I and 6A). Thus, even slight shifts in the relative position of either substrate dramatically reduce the efficiency of peptide bond formation [2],[39]. Indeed, the 2-Å shift of the ester linkage of the P-tRNA observed in the SecM-stalled RNCs would move the carbonyl carbon further away from the A-tRNA (Figures 5I and 6B) and, thus, contribute to the impaired activity of the PTC, explaining the SecM-mediated translational arrest.

### Conclusion

Together with the available biochemistry, our results support a model for SecM stalling in which there are two main contributors to efficient stalling. First, contacts of the SecM nascent chain with the ribosomal tunnel aid positioning of the critical Arg163 of SecM [25] to interact with A2062 of the 23S rRNA [27] (Figure 6B). We believe that this interaction ultimately leads to a shift in the position of the ester linkage of the P-tRNA, which can be a consequence of a direct constraint on the SecM nascent chain and/or can occur through an indirect relay of 23S rRNA nucleotides via A2503 (Figure 6B), as proposed by Vazquez-Laslop et al. [27]. Second, Pro-tRNA^Pro^ in the A-site is critical for stalling [20],[23], as is evident from the observation that the mutation Pro166Ala leads to a reduction in stalling by three orders of magnitude [20],[26],[42]. Therefore, the changed geometry of the PTC appears necessary but not sufficient for stalling. In this respect we note that the strictly required Pro-tRNA^Pro^ in the A-site is characterized by steric constraints and lower nucleophilicity of the N-alkyl amino acid proline [43], compared with the other 19 amino acids. Pro-tRNA^Phe^ is 23-fold slower than Phe-tRNA^Phe^, and Pro-tRNA^bulk^ is 3- to 6-fold slower during peptide bond formation than Ala-tRNA^Ala^ or Phe-tRNA^Phe^ [43], making proline a particularly poor acceptor. Thus, we suggest that the poor chemical properties of proline are exploited to exacerbate the unfavorable geometry of the PTC, leading to efficient translational stalling (Figure 6B). Alternatively, the requirement of Pro-tRNA^Pro^ for stalling could also be explained by the rearrangement at the PTC occurring faster than the rate of peptide bond formation with a proline in the A-site, but slower than that with an alanine. Relief of this conformationally locked inactive state is possible by the residual transferase activity and prolonged incubation time [23] (Figures 1D and 6C), or through the presence of SecA [24]. It is conceivable that the physiological relief provided by the SecA ATPase is triggered by unlocking of the inactive PTC geometry via disruption of SecM interactions with the tunnel. In general, perturbations of the PTC are also evident in other stalling sequences, such as TnaC [4], AAP, and CMV [44], but without a significant shift in the Pro-tRNA, indicating that each stalling sequence appears to utilize a distinct allosteric mechanism.

## Materials and Methods

### Preparation of SecM-Stalled RNCs

The SecM construct was generated by PCR using forward T7_RBS_6xHis (5′-TAATACGACTCACTATAGGGCCTCTAGAAATAATTTTGTTTAACTTTAAGAAGGAGATATACATATGTCTCATCATCATCATCATCAT-3′) ;and reverse DP_SecM (5′-AATGGATTAGTGACAATAAAATTGAATTTACCCCACAAGCAAAATTCAGCACGCCCGTCTGGATAAGCCAGGCGCAAGGCATCCGTGCTGGCCCTCAACGCCTCACCTAATAA-3′) primers with DP120 (without signal anchor) construct as the template (Figure 1A). Uncapped transcripts were then synthesized from the PCR fragments using T7 RNA polymerase. SecM RNCs were generated using an *E. coli* in vitro translation system (Promega) programmed with SecM mRNA.

For in vitro translation, two 500-µl reactions were incubated at 30°C for 20 min (Figure 1B, lane 1). Chloramphenicol (1 µg/ µl) was added to reduce peptidyl-tRNA hydrolysis [40] during the prolonged purification procedure that followed. Each reaction was spun through 500 µl of a high salt sucrose cushion (50 mM HEPES [pH 7.0], 250 mM KOAc, 25 mM Mg[OAc]_2_, 5 mM 2-mercaptoethanol, 0.75 M sucrose, 0.1% Nikkol, 500 µg/ml chloramphenicol, and 0.2 U/ml RNAsin; Promega) and 0.1% pill/ml (1 pill complete protease mix per 1 ml H_2_O; Roche Diagnostics) at 70,000 *g* for 150 min in a TLA 120.2 rotor (Beckman Coulter) at 4°C. The supernatant (Figure 1B, lane 2) was discarded, and the ribosomal pellet (Figure 1B, lane 3) was resuspended in 500 µl of ice-cold 250 buffer (50 mM HEPES [pH 7.0], 250 mM KOAc, 25 mM Mg[OAc]_2_, 5 mM 2-mercaptoethanol, 250 mM sucrose, 0.1% Nikkol, 500 µg/ml chloramphenicol, 0.2 U/ml RNAsin, and 0.1% pill/ml) for 45 min at 4°C, transferred onto 500 µl of Talon Metal Affinity Resin (Clontech) pre-equilibrated with 250 buffer supplemented with 10 µg/ml of tRNAs and incubated for 5 min at room temperature. The resin was washed ten times with 1 ml of ice-cold 250 buffer. RNCs were eluted with 2.5 ml of 250 buffer supplemented with 100 mM imidazole (pH 7.0). The eluted RNCs were spun through 200 µl of a high salt sucrose cushion at 70,000 *g* for 150 min in a TLA 110 rotor at 4°C, and the resulting RNC pellet was resuspended in 1 ml of grid buffer (20 mM HEPES [pH 7.0], 50 mM KOAc, 6 mM Mg[OAc]_2_, 5 mM DTT, 500 µg/ml chloramphenicol, 0.05% Nikkol, 0.5% pill/ml, 0.1 U/ml RNAsin, and 125 mM sucrose) for 30 min at 4°C. The resulting SecM RNC (Figure 1B, lane 4) typically had a yield of approximately 2.5 OD_260_.

An affinity-purified 1 ml of RNCs (2.5 OD_260_) was further applied to 10 ml of sucrose on a 10%–40% gradient in 250 buffer in order to separate the monomeric SecM-stalled RNCs from the polysomes. Gradients were then centrifuged in a Beckman Coulter SW40-Ti rotor at 20,000 rpm for 4 h (4°C). In parallel, 1 ml of crude 70S ribosomes (2.5 OD_260_) prepared from the same extract used for translation was also applied on the sucrose gradient as a control (Figure 1C). The monosome SecM RNC fractions were pooled and concentrated by ultra-centrifugation. The yield of isolated monosome SecM RNCs was typically approximately 0.5 OD_260_. Concentrated monosome SecM RNCs were aliquoted in small volumes, flash frozen in liquid nitrogen, and stored at −80°C until needed.

### Electron Microscopy, Image Processing, and Modeling

As described previously [45], 3.5 µl of SecM RNCs (2.5 OD_260_/ml) was applied to 2-nm carbon-coated holey grids. Micrographs were then recorded under low-dose conditions (25 electrons/Å^2^) with a magnification of 38,900 on a Tecnai F30 field emission gun electron microscope at 300 kV in a defocus range of 1.0–4.0 µm. Micrographs were scanned on a Heidelberg Primescan D8200 drum scanner, resulting in a pixel size of 1.24 Å on the object scale. The data were analyzed by determination of the contrast transfer function using CTFFIND software [46]. The data were further processed with the SPIDER software package [47]. After automated particle picking followed by visual inspection, 1.1 million particles were selected for density reconstruction. The dataset was first sorted semi-supervised into ratcheted (350,000 particles; hybrid A/P- and P/E-t-RNAs) and unratcheted (750,000 particles; A-, P-, and E-tRNAs) sub-datasets [30], using reconstructions of programmed and unprogrammed ribosomes as initial references, respectively (Figure 2). The unratcheted dataset of A-, P-, and E-tRNAs was further sorted into 544,000 particles of P-tRNA, 65,000 particles of A- and P-tRNA, and 40,000 particles of P- and E-tRNA using reconstructions of programmed and unprogrammed ribosomes as references. All sorting steps were performed at a pixel size of 2.44 Å/pixel, and reference volumes were filtered from 15 Å to 20 Å. Sorting processes were continued (normally six to ten rounds of refinement) unless the particle numbers in each sub-dataset reached a constant number, in which case the initial references were offered only in the first round. It is also noteworthy here that at no point was any ratcheted reference used for sorting, and therefore the ratcheted sub-dataset segregated itself from the non-ratcheted sub-dataset in an unsupervised fashion. This clearly indicates that the result of the sorting is indeed due to intrinsic characteristics of the particles and not an artifact due to reference bias.

Densities for the 40S, 60S, and tRNAs were isolated using binary masks. Models were generated as described previously [5], adjusted manually with Coot [48], and minimized with VMD [49]. The CCA-Pro and CCA-Gly positions of the nascent chains were modeled based on an alignment with the *Haloarcula marismortui* 50S subunit in complex with CCA-pcb [39],[50]. Initial docking of X-ray structures of ribosomal particles [8],[11],[12],[51] and cryo-EM maps was performed using Chimera [52], whereas alignment of pdbs utilized PyMol (http://www.pymol.org). All figures were generated using Chimera [52].

### Accession Numbers

The cryo-EM maps of the SecM-stalled RNC and SecM-Pro-RNC have been deposited in EMDataBank (http://www.ebi.ac.uk/pdbe/emdb/) under accession numbers EMD-1829 and EMD-1830, respectively.

## Supporting Information

Figure S1 **Resolution curves for the SecM-stalled RNC subpopulations.** The resolutions of the (A) SecM-stalled RNC, (B) SecM-stalled RNC with A-tRNA, and (C) SecM-Pro-RNC are 5.6 Å, 9.3 Å, and 6.0 Å, respectively, using the 0.5 FSC cutoff criterion.
(0.15 MB TIF)

Figure S2 **Comparison of SecM-stalled RNCs.** Reconstruction of the unsorted SecM-stalled RNC (gray) is compared (in boxed region) with reconstructions of (A) EMD-1143 (yellow) at approximately 15 Å [28], (B) SecM-stalled RNC (orange), (C) SecM-stalled RNC with A-tRNA (green), and (D) SecM-Pro-RNC (cyan), which is ratcheted and contains hybrid A/P- and P/E-tRNAs. Note that there is no observable ratcheting in (A–C), whereas ratcheting of the 30S relative to the 50S is seen in (D). Volumes (B–D) were filtered to 15 Å for comparability with EMD-1143.
(2.55 MB TIF)

Figure S3 **Conformational change in the SecM and SecM-Pro-RNCs.** (A) Top (left) and side (right) views comparing SecM-Pro-RNC (30S, gold; 50S, blue) with SecM-stalled RNC (30S, yellow; 50S, cyan) aligned on the basis of the 50S subunit. Note the inward movement of the L1 stalk towards the P/E-tRNA (green) in the SecM-Pro-RNC as well as the ratcheting of the 30S subunit relative to the 50S. (B and C) Top (left) and side (right) views comparing (B) SecM-stalled RNC (30S, yellow; 50S, cyan) or (C) SecM-Pro-RNC (30S, gold; 50S, blue) with the hybrid state EMD-1541 [34], aligned on the basis of the 50S subunit. Note the similarity in ratcheting between SecM-Pro-RNC and EMD-1541.
(3.17 MB TIF)

Figure S4 **Visualization of the SecM nascent chain in the SecM-stalled RNC.** (A–C) Transverse sections through (A) SecM-stalled RNC (gray, with SecM-tRNA in green) at 5.6 Å, (B) EMD-1143 (yellow) at approximately 15 Å [28], and (C) SecM-stalled RNC (orange) filtered to 15 Å. All volumes were set at the same threshold. (D) Comparison of (A) and (B). (E) Comparison of (B) and (C). (F) Comparison of (A) and (C).
(1.31 MB TIF)

Figure S5 **Comparison of SecM and TnaC nascent chains within the tunnel.** (A and B) Transverse sections through (A) SecM-stalled RNC (nascent chain density in green mesh, with model in ribbon with balls for Cα atoms) and (B) TnaC-stalled RNC [4] (nascent chain density in orange mesh, with model in ribbon with Ala sidechains). (C) Comparison of molecular models from (A) and (B). The rRNA is shown as gray surface, with ribosomal proteins L4 (purple), L22 (red), and L23 (yellow) highlighted.
(2.52 MB TIF)

Figure S6 **Comparison of SecM-stalled RNC filtered to different resolutions.** Transverse sections through SecM-stalled RNC with SecM-tRNA in green and mRNA in red, filtered to (A) 6–7 Å, (B) 8–9 Å, and (C) 9–10 Å. Note the presence of small remaining density for the nascent chain in the upper tunnel but predominantly at the lower tunnel at 8–9 Å (B), indicating regions of compaction [5].
(1.19 MB TIF)

Figure S7 **Comparison of PTC of SecM-stalled and non-stalling peptide RNCs.** Views of the PTC of the SecM-stalled-RNC alone (A and D) or compared with TnaC-stalled RNC (B and E) [4], or *E. coli* RNC with a non-stalling peptide at 7.1 Å (0.5 FSC) resolution (C and F) (generated using a truncated mRNA; J. Frauenfeld and R. Beckmann, unpublished data). Density for the SecM-stalled RNC is shown as gray surface in (A) and gray mesh in (D–F), with the model for the SecM-tRNA in green. Densities for the TnaC-stalled and non-stalling peptide RNCs are shown as yellow and blue surfaces in (B and E) and (C and F), respectively, with the molecular models for the peptidyl-tRNAs in gold and dark blue, respectively.
(1.76 MB TIF)

Table S1 **Comparison of interactions of SecM and TnaC nascent chains with components of the ribosomal tunnel.**
(0.06 MB DOC)

## Abbreviations

aa: amino acid
EM: electron microscopy
FSC: Fourier shell correlation
PTC: peptidyl transferase center
RNC: ribosome nascent chain complex
rRNA: ribosomal RNA
