# Supplementary figures and images for: SecM-Stalled Ribosomes Adopt an Altered Geometry at the Peptidyl Transferase Center
Source: PLoS Biol. 2011 Jan 18;9(1):e1000581. doi: 10.1371/journal.pbio.1000581 (PMC3022528; doi:10.1371/journal.pbio.1000581)

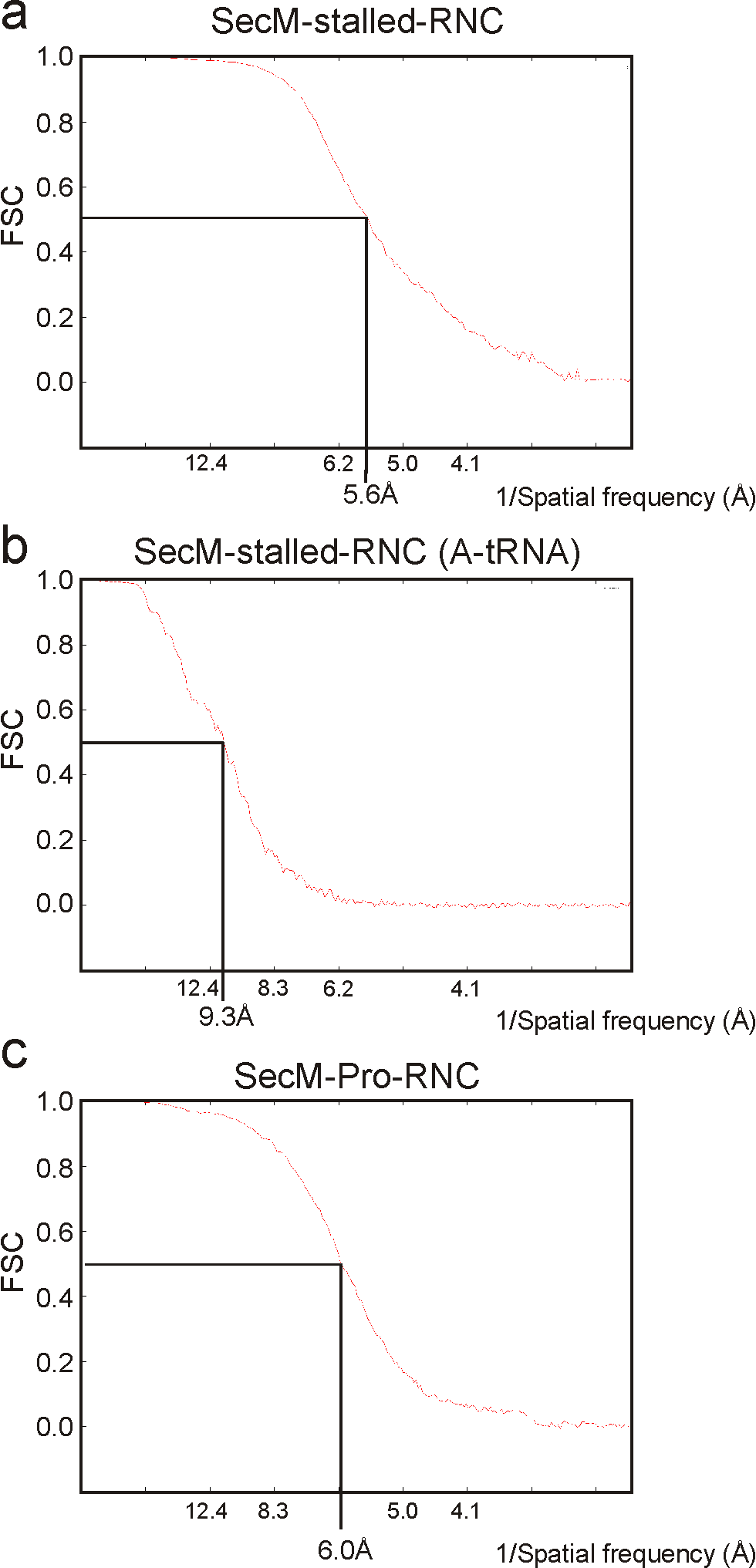

Supplement: Figure S1 — Resolution curves for the SecM-stalled RNC subpopulations. The resolutions of the (A) SecM-stalled RNC, (B) SecM-stalled RNC with A-tRNA, and (C) SecM-Pro-RNC are 5.6 Å, 9.3 Å, and 6.0 Å, respectively, using the 0.5 FSC cutoff criterion. (0.15 MB TIF) [file pbio.1000581.s001.tif]

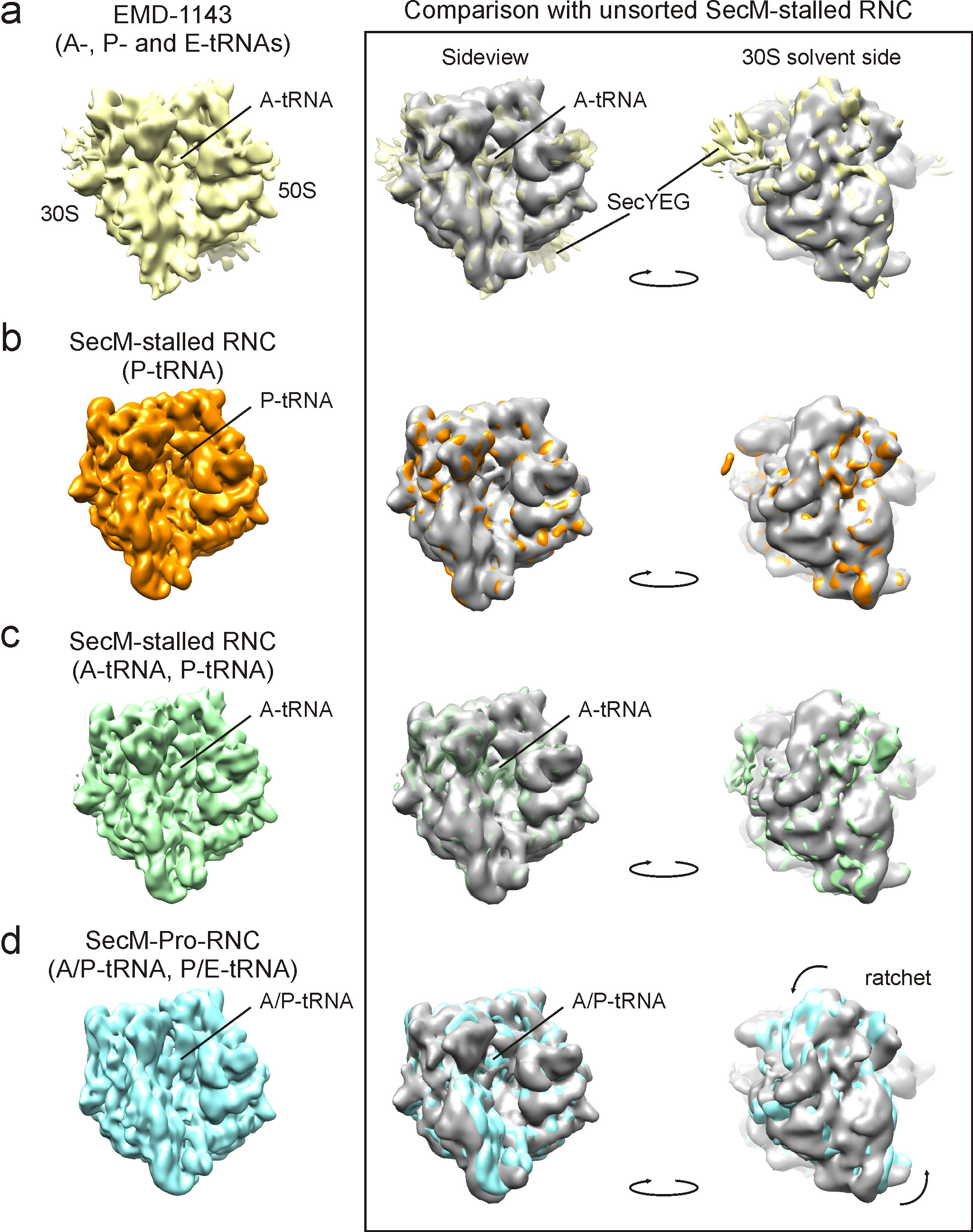

Supplement: Figure S2 — Comparison of SecM-stalled RNCs. Reconstruction of the unsorted SecM-stalled RNC (gray) is compared (in boxed region) with reconstructions of (A) EMD-1143 (yellow) at approximately 15 Å [28], (B) SecM-stalled RNC (orange), (C) SecM-stalled RNC with A-tRNA (green), and (D) SecM-Pro-RNC (cyan), which is ratcheted and contains hybrid A/P- and P/E-tRNAs. Note that there is no observable ratcheting in (A–C), whereas ratcheting of the 30S relative to the 50S is seen in (D). Volumes (B–D) were filtered to 15 Å for comparability with EMD-1143. (2.55 MB TIF) [file pbio.1000581.s002.tif]

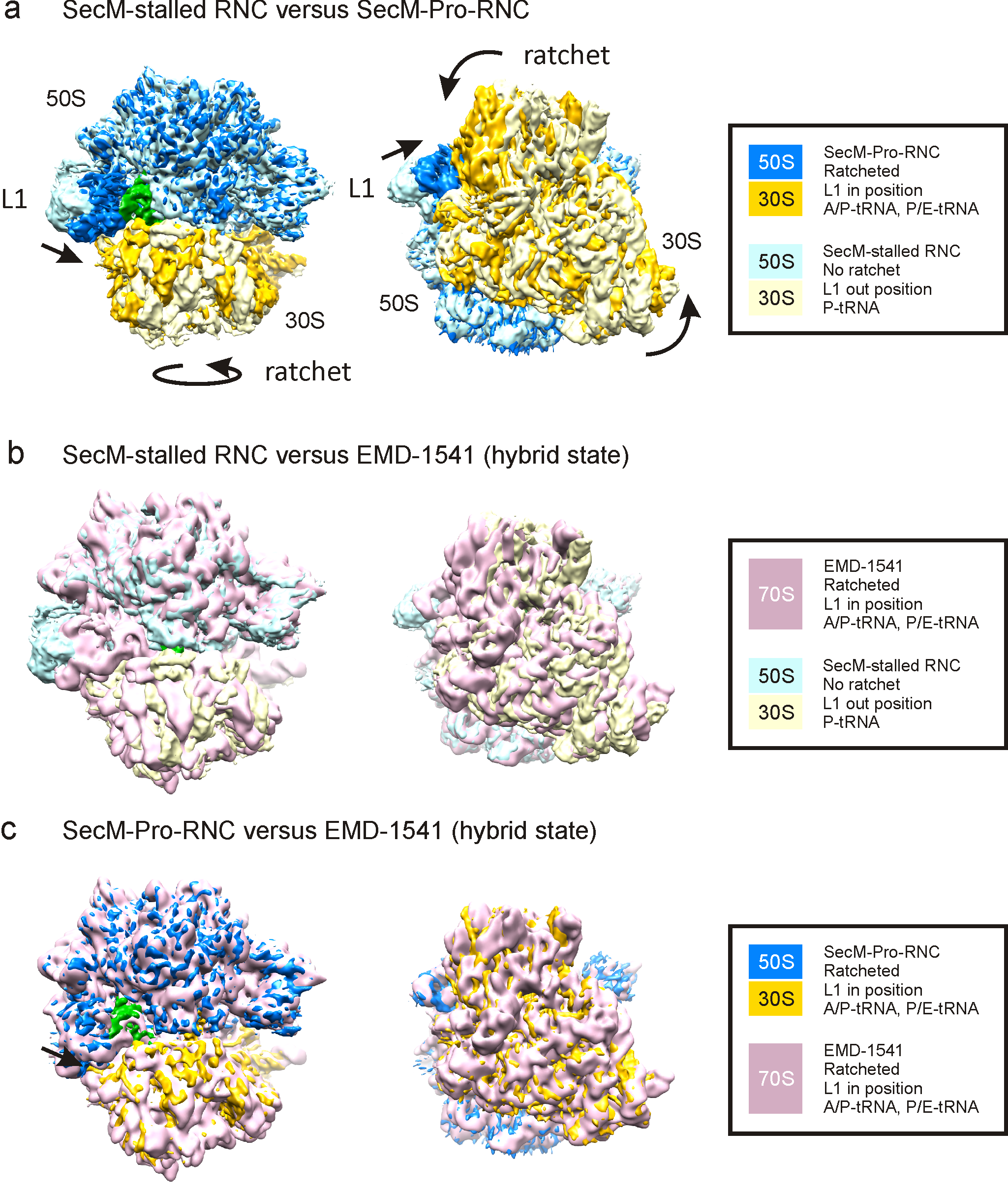

Supplement: Figure S3 — Conformational change in the SecM and SecM-Pro-RNCs. (A) Top (left) and side (right) views comparing SecM-Pro-RNC (30S, gold; 50S, blue) with SecM-stalled RNC (30S, yellow; 50S, cyan) aligned on the basis of the 50S subunit. Note the inward movement of the L1 stalk towards the P/E-tRNA (green) in the SecM-Pro-RNC as well as the ratcheting of the 30S subunit relative to the 50S. (B and C) Top (left) and side (right) views comparing (B) SecM-stalled RNC (30S, yellow; 50S, cyan) or (C) SecM-Pro-RNC (30S, gold; 50S, blue) with the hybrid state EMD-1541 [34], aligned on the basis of the 50S subunit. Note the similarity in ratcheting between SecM-Pro-RNC and EMD-1541. (3.17 MB TIF) [file pbio.1000581.s003.tif]

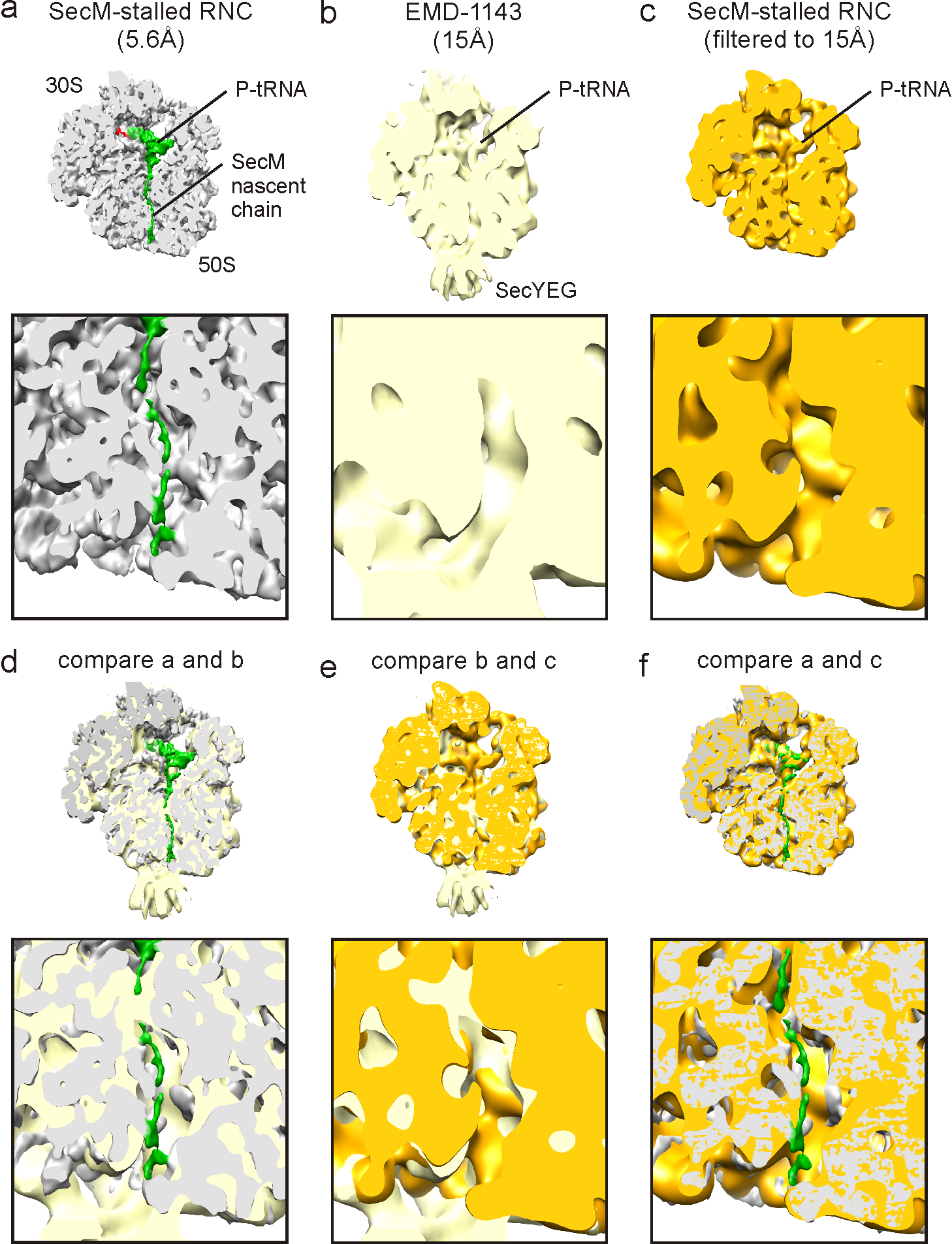

Supplement: Figure S4 — Visualization of the SecM nascent chain in the SecM-stalled RNC. (A–C) Transverse sections through (A) SecM-stalled RNC (gray, with SecM-tRNA in green) at 5.6 Å, (B) EMD-1143 (yellow) at approximately 15 Å [28], and (C) SecM-stalled RNC (orange) filtered to 15 Å. All volumes were set at the same threshold. (D) Comparison of (A) and (B). (E) Comparison of (B) and (C). (F) Comparison of (A) and (C). (1.31 MB TIF) [file pbio.1000581.s004.tif]

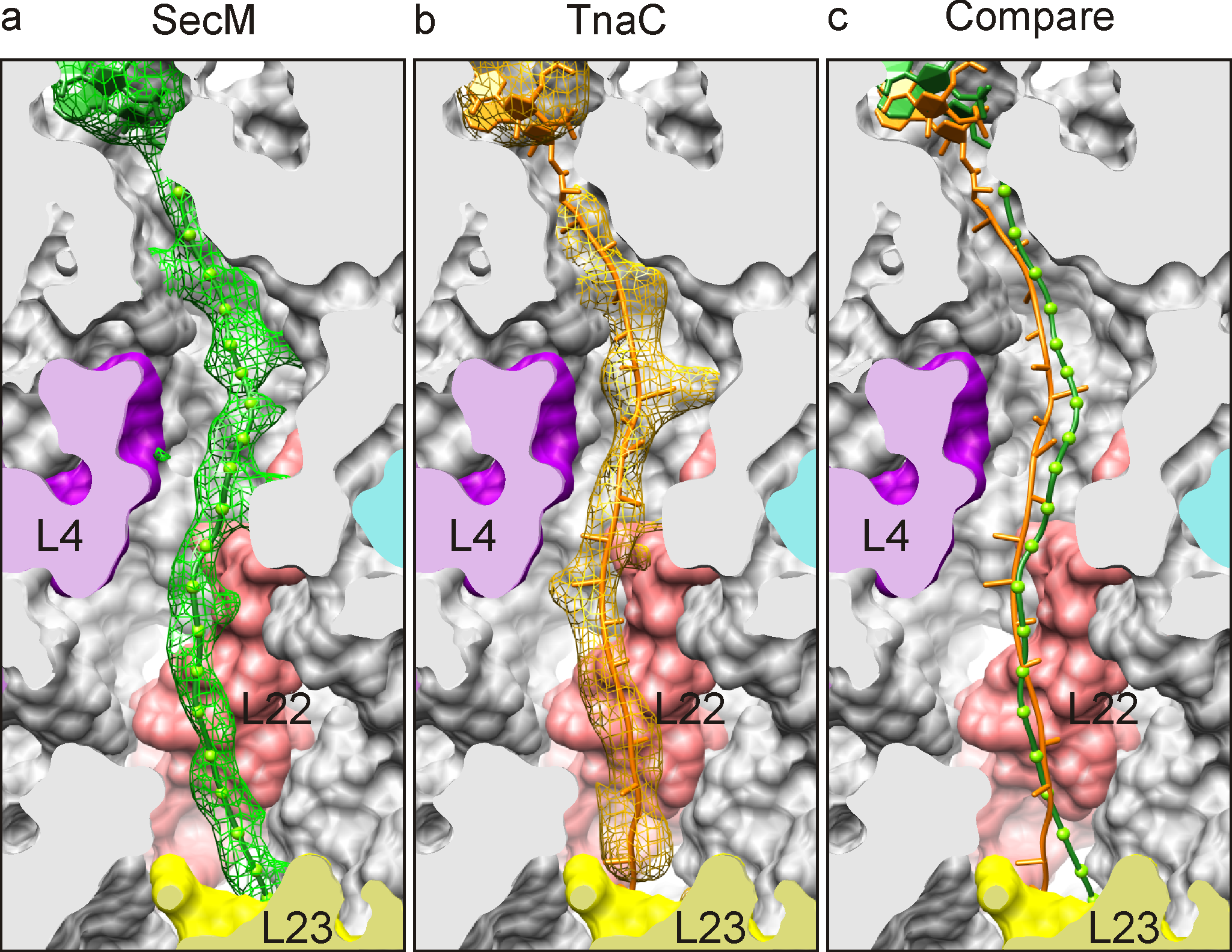

Supplement: Figure S5 — Comparison of SecM and TnaC nascent chains within the tunnel. (A and B) Transverse sections through (A) SecM-stalled RNC (nascent chain density in green mesh, with model in ribbon with balls for Cα atoms) and (B) TnaC-stalled RNC [4] (nascent chain density in orange mesh, with model in ribbon with Ala sidechains). (C) Comparison of molecular models from (A) and (B). The rRNA is shown as gray surface, with ribosomal proteins L4 (purple), L22 (red), and L23 (yellow) highlighted. (2.52 MB TIF) [file pbio.1000581.s005.tif]

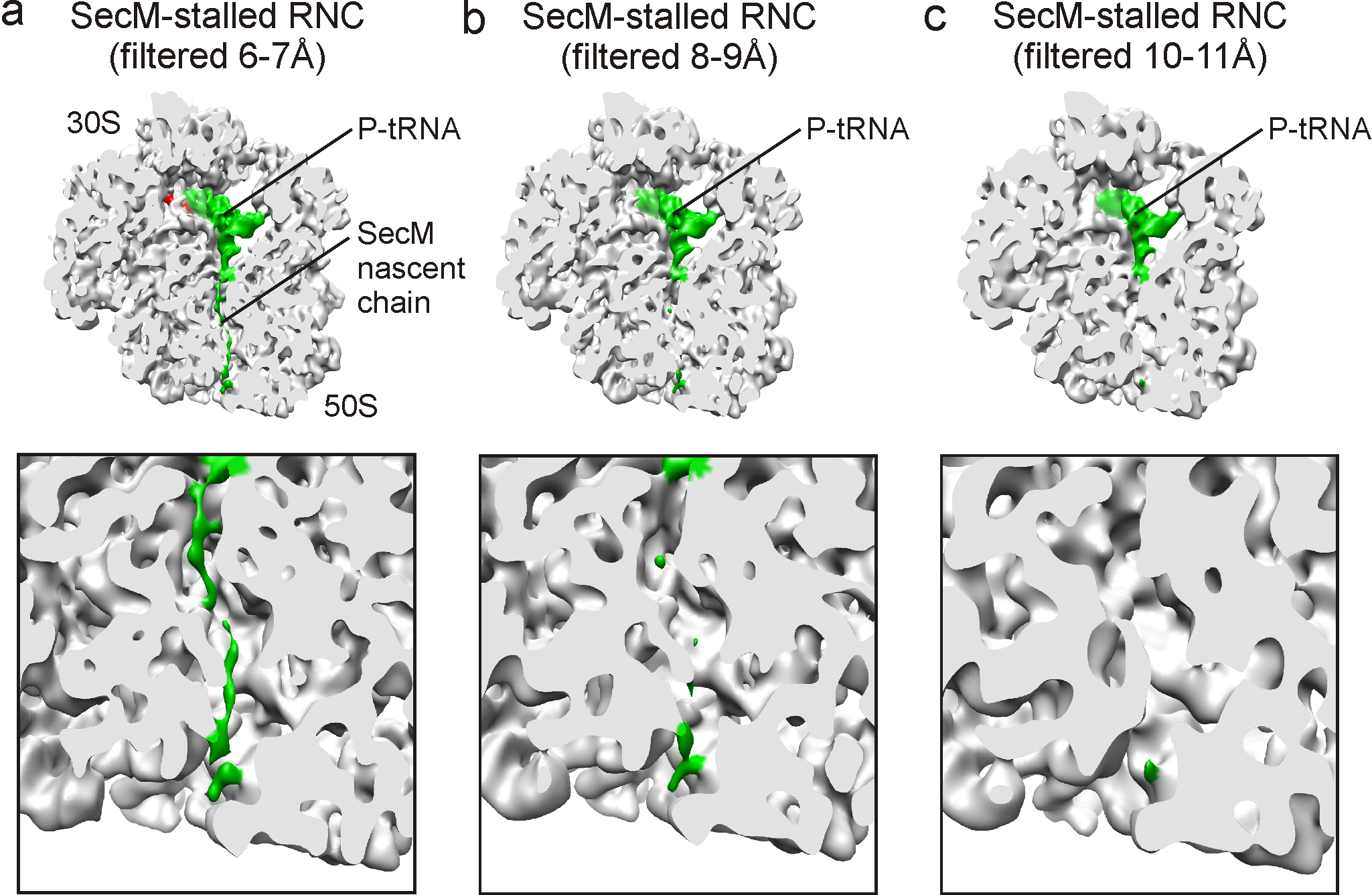

Supplement: Figure S6 — Comparison of SecM-stalled RNC filtered to different resolutions. Transverse sections through SecM-stalled RNC with SecM-tRNA in green and mRNA in red, filtered to (A) 6–7 Å, (B) 8–9 Å, and (C) 9–10 Å. Note the presence of small remaining density for the nascent chain in the upper tunnel but predominantly at the lower tunnel at 8–9 Å (B), indicating regions of compaction [5]. (1.19 MB TIF) [file pbio.1000581.s006.tif]

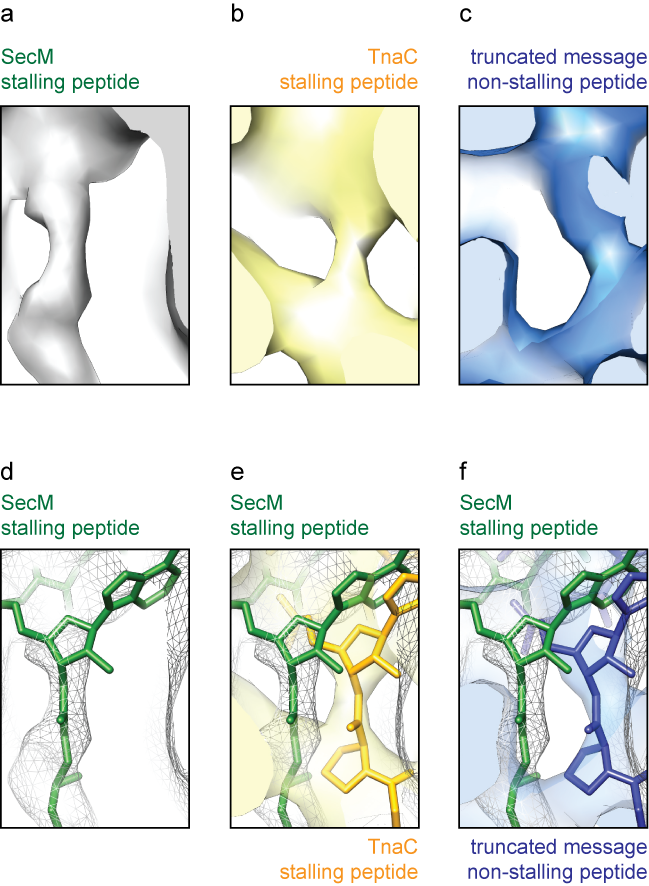

Supplement: Figure S7 — Comparison of PTC of SecM-stalled and non-stalling peptide RNCs. Views of the PTC of the SecM-stalled-RNC alone (A and D) or compared with TnaC-stalled RNC (B and E) [4], or E. coli RNC with a non-stalling peptide at 7.1 Å (0.5 FSC) resolution (C and F) (generated using a truncated mRNA; J. Frauenfeld and R. Beckmann, unpublished data). Density for the SecM-stalled RNC is shown as gray surface in (A) and gray mesh in (D–F), with the model for the SecM-tRNA in green. Densities for the TnaC-stalled and non-stalling peptide RNCs are shown as yellow and blue surfaces in (B and E) and (C and F), respectively, with the molecular models for the peptidyl-tRNAs in gold and dark blue, respectively. (1.76 MB TIF) [file pbio.1000581.s007.tif]
