# Supplementary material for: SecM-Stalled Ribosomes Adopt an Altered Geometry at the Peptidyl Transferase Center
Source: PLoS Biol. 2011 Jan 18;9(1):e1000581. doi: 10.1371/journal.pbio.1000581 (PMC3022528; doi:10.1371/journal.pbio.1000581)
Supplement: Table S1 — Comparison of interactions of SecM and TnaC nascent chains with components of the ribosomal tunnel. (0.06 MB DOC) [file pbio.1000581.s008.doc]

**Supplemental Table 1** Comparison of interactions of SecM and TnaC nascent chains with components of the ribosomal tunnel

| **SecM nascent  chain residue*^1^** | | **Ribosomal tunnel components** | | **TnaC nascent  chain residue*^1^** | |
| --- | --- | --- | --- | --- | --- |
| 0 | Gly165 | **U2585** | | Pro24 | 0 |
| -1 | Ala164 | - | G2061, G2505-U2506 | Arg23 | -1 |
| -2 | Arg163 | **A2062** | | His22 | -2 |
| -3 | Ile162 | - | U2586/U1782 | Asp21 | -3 |
| -4 | Gly161 | - | - | Val20 | -4 |
| -5 | Gln160 | - | A2058-A2059 | Ile19 | -5 |
| -6 | Ala159 | - | U2609 | Lys18 | -6 |
| -7 | Gln158 | **(A752)** | **A752** |  |  |
| -8 | Ser157 | - | A2058-A2059 | Asp16 | -8 |
|  |  | - | U747 |  |  |
| -9 | Ile156 | **(L4: Lys63-Thr65)** | **L4: Lys63-Thr65** | Asn14 | -10 |
| -10 | Trp155 | **A751** | | Phe13 | -11 |
|  |  | **(L22: Lys90-Arg92)** | **L22: Lys90-Arg92** | Trp12 | -12 |
| -12 | Pro153 | - | (L4: Arg67) | Lys11 | -13 |
| -13 | Thr152 | (L22: 90-91) |  |  | -14 |
| -14 | Ser151 | - | L22: Arg95 | Thr9 | -15 |
| -15 | Phe150 | (C461) |  |  | -16 |
| -16 | Lys149 | **(L22: Met82-Arg84)** | | Cys7 | -17 |
| -17 | Gln147 | **(L23: Gln72)** | | His5 | -19 |
| -18 | Pro146 | - | (L22: His9) | Leu4 | -20 |
| -20 | Phe144 | **A1321 (in H50)** | | Asn2 | -22 |

*^1^Positions are approximate so the closest residue(s) to the contact site are given, based on fitting of SecM nascent chain to cryo-EM density

*^2^Positions are approximate, but the closest components to the contact are given based on fitting of *E. coli* 70S crystal structure to SecM-70S cryo-EM density.

*^3^Residues of the nascent chain that have been implicated in SecM or TnaC-mediated stalling are highlighted in gray.
